# The value of usability testing for Internet-based adolescent self-management interventions: “Managing Hemophilia Online”

**DOI:** 10.1186/1472-6947-13-113

**Published:** 2013-10-04

**Authors:** Vicky R Breakey, Ashley V Warias, Danial M Ignas, Meghan White, Victor S Blanchette, Jennifer N Stinson

**Affiliations:** 1 Division of Hematology/Oncology, Department of Pediatrics, McMaster Children’s Hospital, 1200 Main Street West, Hamilton, Ontario, L8N 3Z5, Canada; 2 Faculty of Health Sciences, McMaster University, 1280 Main Street West, Hamilton, Ontario, L8S 4K1, Canada; 3 Child Health Evaluative Sciences, The Hospital for Sick Children Research Institute, 555 University Avenue, Toronto, Ontario, M5G 1X8, Canada; 4 Department of Pediatrics, The Hospital for Sick Children, 555 University Avenue, Toronto, Ontario, M5G 1X8, Canada; 5 Lawrence S. Bloomberg Faculty of Nursing, University of Toronto, 155 College Street, Suite 130, Toronto, Ontario, M5T 1P8, Canada; 6 Department of Anesthesia and Pain Medicine, The Hospital for Sick Children, 555 University Avenue, Toronto, Ontario, M5G 1X8, Canada

**Keywords:** Usability testing, Internet, Patient education, Self-management, Adolescent, Hemophilia

## Abstract

**Background:**

As adolescents with hemophilia approach adulthood, they are expected to assume responsibility for their disease management. A bilingual (English and French) Internet-based self-management program, “Teens Taking Charge: Managing Hemophilia Online,” was developed to support adolescents with hemophilia in this transition. This study explored the usability of the website and resulted in refinement of the prototype.

**Methods:**

A purposive sample (*n*=18; age 13–18; mean age 15.5 years) was recruited from two tertiary care centers to assess the usability of the program in English and French. Qualitative observations using a “think aloud” usability testing method and semi-structured interviews were conducted in four iterative cycles, with changes to the prototype made as necessary following each cycle. This study was approved by research ethics boards at each site.

**Results:**

Teens responded positively to the content and appearance of the website and felt that it was easy to navigate and understand. The multimedia components (videos, animations, quizzes) were felt to enrich the experience. Changes to the presentation of content and the website user-interface were made after the first, second and third cycles of testing in English. Cycle four did not result in any further changes.

**Conclusions:**

Overall, teens found the website to be easy to use. Usability testing identified end-user concerns that informed improvements to the program. Usability testing is a crucial step in the development of Internet-based self-management programs to ensure information is delivered in a manner that is accessible and understood by users.

## Background

Hemophilia is a bleeding disorder that results from a deficiency of coagulation factor VIII (Hemophilia A) or IX (Hemophilia B) [1]. It is treated with intravenously administered replacement factor that is often given at home. Despite factor replacement, individuals with hemophilia often bleed into joints and muscles, necessitating additional infusions, pain management and rehabilitation.

Like teens with other chronic conditions, adolescents with hemophilia (AWH) must become competent in managing their disease. The complex biological, social and emotional changes inherent in adolescence make this increasingly challenging for many teens [2]. A recent study showed that at a median age of 17.2 years, almost 25% of AWH still required parental assistance in hemophilia-related care [3]. It is essential that health care providers find effective means of filling knowledge gaps and encouraging the development of self-management skills in adolescents with chronic illnesses.

The Internet has emerged as a primary means of communication and information delivery [4]. The appeal of social networking and virtual tools to adolescents has fostered the development of a technologically-savvy demographic that is proficient at accessing information online [5]. Recently, several Internet-based educational interventions have been developed to meet the needs of adolescents with chronic diseases [6]. Formal evaluations of these programs have revealed their capacity to enhance the availability, accessibility and acceptability of educational programs in this patient population, citing the Internet as a highly appealing and well-suited medium for the delivery of self-management education to adolescents [6]. Presently, there are no documented self-management programs for AWH available online.

We developed an online intervention to meet the educational needs of AWH, modeled after a similar online educational program for teens with juvenile idiopathic arthritis [7]. Prior to studying the efficacy of the intervention, we embarked on usability testing of the website to ensure appropriate ease of use (navigation), comprehension and satisfaction. Usability assessment has been well-studied in the implementation of clinical information systems and has been shown to be critical to their success [8]. For internet-based health interventions, usability testing is essential to ensure that patients’ needs are met and that they are able to navigate the website appropriately and efficiently [9]. In general, usability testing consists of users assessing the website prototype in iterations, with changes implemented as necessary following each iteration with the goal of improving the product. Recent medical literature reviews and compares usability testing methods used to assess health technologies [8,10].

This study implemented a user-based approach to assess the usability of a newly developed online self-management program, “Teens Taking Charge: Managing Hemophilia Online.” The online hemophilia self-management intervention was assessed in terms of (1) user performance, including ease of use and user efficiency (as measured by observed navigation) and (2) participants’ satisfaction (program likes and dislikes as measured by a semi-structured interview). Our methodology and the changes adopted following testing give insight into the value of usability testing for online patient education interventions.

## Methods

### Website development

**Figure 1:**
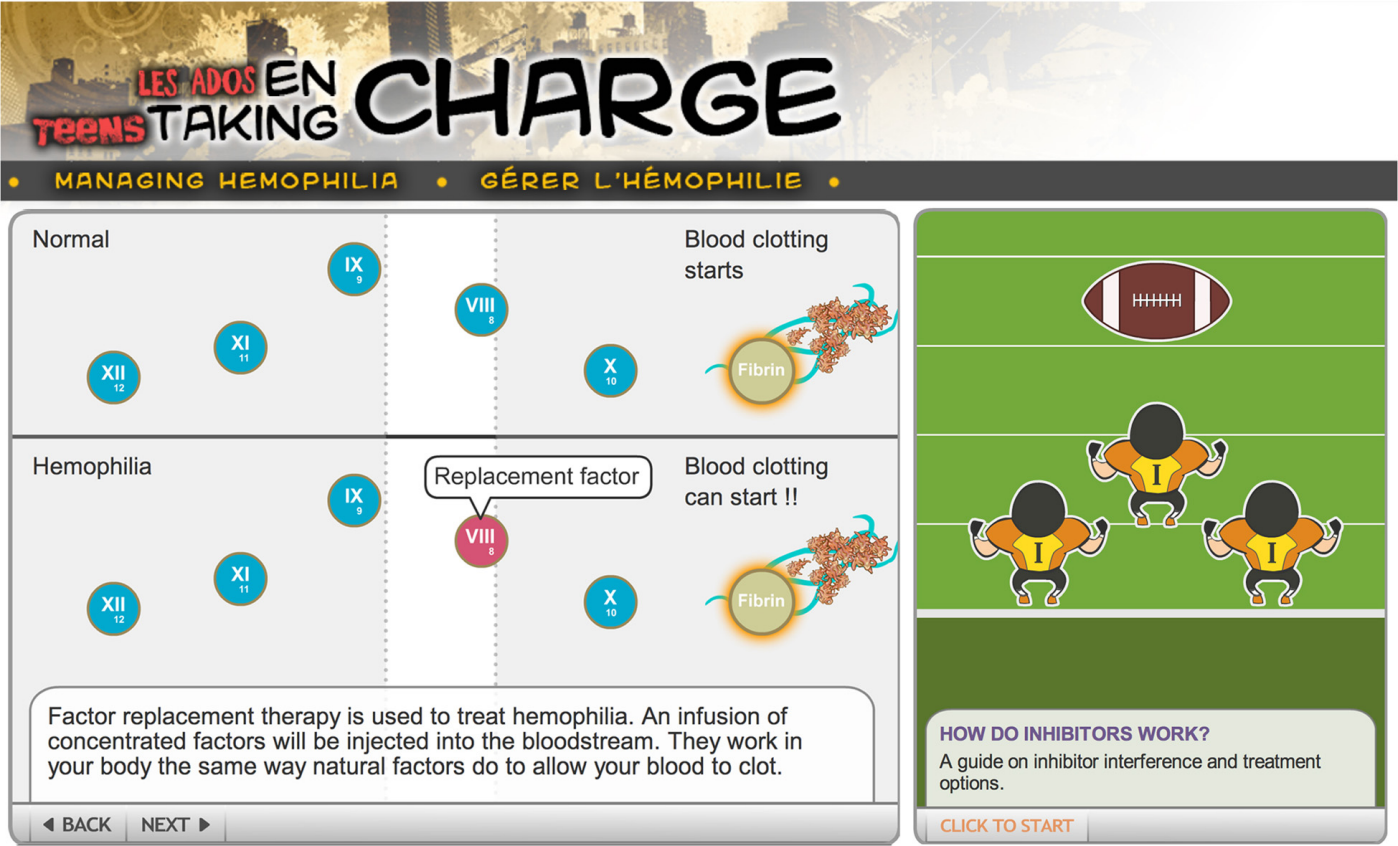
**Examples of interactive content found on the website.** Left: An animation illustrating blood clotting and the role of factor replacement therapy. Right: An animation using an analogy comparing inhibitors to defensive football players.

“Teens Taking Charge: Managing Hemophilia Online” is an interactive website designed to assist AWH in their transition from pediatric to adult care (Figure 1). Following an in-depth needs assessment of AWH [11-13], content was developed by a team of Anglophone and Francophone health care experts and AWH from across Canada. Content was written at the suggested grade 6–7 reading level for patient education materials [14]. The website is comprised of 80 web pages of content and includes >35, 000 words. Content is organized into eight modules that include hemophilia-specific education, self-management strategies, images, interactive animations, quizzes and a glossary (Table 1). Content was developed in English and translated into French. Translated content was reviewed by Francophone health care experts for accuracy.

**Table 1:** The eight content modules of the website

**Program modules:**	
**I**	About the Program
**II**	The Basics of Hemophilia
**III**	Hemophilia Management
**IV**	Managing Bleeds
**V**	Complications of Hemophilia
**VI**	Hemophilia: Mind and Body
**VII**	Transition of Care
**VIII**	Looking Ahead

### Participants

Adolescents were recruited from the hematology clinics of two large pediatric tertiary care centers: The Hospital for Sick Children (SickKids), Toronto, and CHU Sainte-Justine, Montreal. As the largest English and French speaking centres in Canada, these sites were felt to provide good representation of target end-users of the program. The study was approved by both The Hospital for Sick Children’ and CHU Sainte-Justine’s Research Ethic Boards.

Adolescents were eligible to participate if (1) they were 13 to 18 years of age, (2) they had been diagnosed with mild, moderate, or severe hemophilia A or B and (3) they could speak and read either English or French fluently. Potential participants were excluded if they had major cognitive impairments or if a member of their health care team indicated that they had a co-morbid medical or psychiatric condition that was likely to impact their ability to participate.

### Usability methodology

A qualitative usability testing approach with audio-taped observation and semi-structured interviews by a trained observer was conducted in iterative cycles to determine the usability and intuitiveness of the user interface of the Internet-based hemophilia self-management intervention and to further refine the prototype. The methodology employed a “think aloud” approach that is user-based that aims to gather insight into the way users solve problems encountered during website usage [8]. This method occurs in two steps: 1) collecting think aloud data in a systematic way and 2) analyzing the data to obtain a modle of the cognitive processes that occur as the user addresses a problem [15]. Practically, experts walk users through the prototype and observe their approach to assigned tasks to provide information about the usability of the website [8]. Participants were encouraged to think aloud and comments are recorded, transcribed and analyzed qualitatively.

Usability testing produces informative results with small sample sizes [9]. Nielsen’s law of diminishing returns suggests that 80% of usability problems can be identified with four or five subjects and 95% of problems identified with nine participants [16,17]. Iterative rounds of testing with 4–6 adolescents per round were conducted until data saturation was achieved.

### Study procedure

After obtaining written informed consent for study participation and audio-taping, participants were asked to complete two brief questionnaires that collected basic demographic, health and Internet-use characteristics (Additional file 1). Adolescents then participated in a one-on-one observation, followed by a semi-structured interview with a research assistant (RA). The RA first directed the participant to the main navigation menu and prompted them to select one of the modules that was of interest to them. Once in the module, the RA, working from a standardized script, directed the participant to one or more key features within that module. This continued until the participant had viewed all of the modules or showed disinterest in exploring further. Adolescents were encouraged to adopt a “think aloud” approach as they navigated through the website . The RA observed participants and took field notes on any difficulties encountered during testing. While interviews were projected to take 45–60 minutes, length varied depending on the participants’ clinic schedules and interest, with the longest interview lasting approximately 90 minutes and the shortest 20 minutes.

The usability of the website was assessed in terms of user performance and satisfaction. User performance was observed as participants navigated through standardized website content and features. Any navigation difficulties or errors were recorded. User satisfaction was assessed based on participant’s responses (likes and dislikes) to the content and web interface throughout the semi-structured interview that included set of standardized questions to assess specific components and their overall impression of the site (Table 2).

**Table 2:** Standardized questions asked of participants at the interview close

**Post-usability question set:**	
**1.**	What was your overall impression of “Teens Taking Charge: Managing Hemophilia” online?
	a. What is your impression of “the look” of the site?
	a. What did you think about the animations? Illustrations? Videos?
	a. How easy was it to use/navigate?
**2.**	What did you like about the website?
	a. Why?
**3.**	What do you think could be improved?
	b. How could this/these things be improved?
**4.**	Did you think there was anything missing from the website?
	c. How could we better address this area?

Following each iteration, data was analyzed by three co-investigators (VB, DI and AW) and changes were made to the website. Subsequent to each cycle of revisions, additional iterations of usability testing were conducted until no significant issues were identified.

### Data analysis

Quantitative data from participant questionnaires was analyzed using Microsoft Excel 2008 to show descriptive statistics, including measures of central tendency and frequencies. Audio-tapes from interviews were transcribed verbatim. French interviews were transcribed directly into English by a bilingual RA (MW). Field notes were incorporated into transcripts.

Simple qualitative content analysis, as outlined by Sandelowski [18], was used to analyze transcripts after each iteration. Following the first iteration, transcripts were read independently by three investigators (VB, AW and DI) and coding was developed to describe the data thematically. Disagreements (e.g. wording of themes) were handled through consensus of the analysts. Resulting usability issues were relayed to the website development team who made changes to the website prototype. Following each successive iteration, all new data was entered into the analysis under the existing themes where appropriate and new themes were generated as necessary until there was no new data that could not be categorized under the existing codes [19]. No further modifications to the website were made after the fourth round of testing.

## Results

### Participant characteristics

Patients meeting the study criteria were recruited by a member of the research team. Among eligible individuals, approximately 20% of teens approached refused participation. Eighteen adolescents agreed to participate. The mean age of the sample was 15.5 years. Sixty-seven percent of the sample had hemophilia A, 22% hemophilia B, and 11% were unsure of their hemophilia type. Seventeen percent of participants described their disease severity as mild, 17% moderate and 67% severe. Nearly all participants were high-school students. Participants’ complete demographic and health data are shown in Table 3.

**Table 3:** Demographic and health characteristics of adolescents from iterative cycles 1–4

**Characteristic**		**English cycle 1 (n=4)**	**English cycle 2 (n=4)**	**English cycle 3 (n=4)**	**French cycle 4 (n=6)**
**Hemophilia Type, n (%)**					
A		1 (25%)	4 (100%)	2 (50%)	5 (83%)
B		2 (50%)		2 (50%)	
Unsure		1 (25%)			1 (17%)
**Disease Severity, n (%)**					
Mild		2 (50%)		1 (25%)	
Moderate			1 (25%)	1 (25%)	1 (17%)
Severe		2 (50%)	3 (75%)	2 (50%)	5 (83%)
**Currently on Prophylaxis, n (%)**		2 (50%)	4 (100%)	2 (50%)	6 (100%)
**Age, n (%)**					
13			1 (25%)	1 (25%)	
14		2 (50%)	1 (25%)	1 (25%)	
15					1 (17%)
16		1 (25%)		1 (25%)	3 (50%)
17		1 (25%)	2 (50%)	1 (25%)	2 (33%)
18					
**Grade in School, n (%)**			2 (50%)		
**English**	**French**				
7–8	1–2				
9–12	3–5	4 (100%)	2 (50%)	4 (100%)	6 (100%)
>12	CEGEP				

All participants had a computer and access to the Internet at home. All participants stated that they used the computer and Internet on a weekly basis and two-thirds of the sample spent 5 or more hours per week online. Majority stated they felt very comfortable using the computer and the Internet. Table 4 provides complete statistics on participants’ computer and Internet use.

**Table 4:** Computer and Internet use by adolescents from iterative cycles 1–4

**Characteristic**	**English cycle 1 (n=4)**	**English cycle 2 (n=4)**	**English cycle 3 (n=4)**	**French cycle 4 (n=6)**
**Computer at home, n (%)**				
Yes	4 (100%)	4 (100%)	4 (100%)	6 (100%)
No				
**Internet at home, n (%)**				
Yes	4 (100%)	4 (100%)	4 (100%)	6 (100%)
No				
**Computer at school, n (%)**				
Yes	4 (100%)	3 (75%)	4 (100%)	6 (100%)
No		1 (25%)		
**Hours spent on computer per week, n (%)**				
0				
1–2	1 (25%)	1 (25%)	1 (25%)	1 (17%)
2–3		1 (25%)		1 (17%)
3–4				
4–5		1 (25%)		1 (17%)
5–6	1 (25%)		1 (25%)	
6–7			1 (25%)	1 (17%)
≥7	2 (50%)	1 (25%)	1 (25%)	2 (33%)
**Comfort level on computer**				
Not at all comfortable				
A little comfortable				1 (17%)
Comfortable	1 (25%)	1 (25%)	1 (25%)	3 (50%)
Very comfortable	3 (75%)	3 (75%)	3 (75%)	2 (33%)
**Hours spent on Internet per week, n (%)**				
0				
1–2	1 (25%)			1 (17%)
2–3		2 (50%)	1 (25%)	1 (17%)
3–4			1 (25%)	
4–5				1 (17%)
5–6	1 (25%)		1 (25%)	
6–7				1 (17%)
>7	2 (50%)	2 (50%)	1 (25%)	2 (33%)
**Comfort level on Internet**				
Not at all comfortable				
A little comfortable				2 (33%)
Comfortable	1 (25%)		1 (25%)	2 (33%)
Very comfortable	3 (75%)	4 (100%)	3 (75%)	2 (33%)

### Website usability

Four cycles involving 18 participants total were necessary. Due to logistical limitations, the first, second and third iterations were conducted at the first testing site with four English-speaking adolescents per round and the fourth iteration of testing occurred at the second testing site with 6 French-speaking adolescents. For all iterations, convenience sampling based on clinic scheduling of potential subjects was implemented to achieve a representative sample with respect to age and disease severity. Significant changes made following each cycle of testing can be found in Table 5.

**Table 5:** Summary of problems cited by participants and subsequent changes implemented

		
	**Problems cited**	**Changes implemented**
**Iteration 1 English, n=4**	A Venn diagram (Figure 2) comparing and contrasting the symptoms of arthritis and joint bleeds was not well understood by three users.	The Venn diagram was re-tested in the second iteration to better identify the nature of users’ confusion.
	Two users found the background colouring of the “History of Hemophilia” animation too dark and had difficulty understanding how to move through the animation. Users also suggested the animation be enlarged.	The background colouring of the “History of Hemophilia” animation was lightened and instructions for use were included (Figure 6). The animation was also doubled in size.
	Text explaining hemostasis, through an analogy to road repair, was found difficult to follow by users.	The text was reproduced as a table (Figure 8).
	Author credentials listed at the bottom of each web page were not understood by participants.	Credentials were replaced with a brief lay sentence explaining each author’s professional role.
	Two users sought a control to enlarge video size.	The content management system did not support enlarged video.
	All participants experienced difficulty navigating between modules and returning to the website’s home page.	Text in the navigation menu was modified to change colour when clicked upon. A “home” button was added to the navigation menu.
	Two users were unfamiliar with the mnemonic RICE, which was used as a caption for an image depicting bleed management.	The mnemonic was re-tested in the second iteration to determine whether a higher proportion of users were unfamiliar with its meaning.
	To emphasize the fragility of the synovial capsule, an analogy to a water balloon was made. Two users did not understand the analogy.	A caption explaining the purpose of the analogy was included below a side-by-side image of a synovial capsule and water balloon.
	**Problems cited**	**Changes implemented**
**Iteration 2 English, n=4**	An image illustrating the X-linked inheritance pattern of hemophilia (Figure 4) did not clearly discern why an affected father could not pass the trait onto his male offspring.	Maternal and paternal chromosomes were differentially coloured to illustrate that offspring derive one sex chromosome from each parent (Figure 5).
	The Venn diagram (Figure 2) continued to pose comprehension difficulties for users.	The Venn diagram was broken down into multiple figures to first illustrate the symptoms of joint bleeds and arthritis as distinct sets, and then depict their joining (Figure 3).
	Users continued to find the background colouring of the “History of Hemophilia” animation inadequate.	The “History of Hemophilia” animation was re-tested in the third iteration to expand upon user suggestions for improvement.
	Two additional users were unfamiliar with the mnemonic RICE. Users familiar with the mnemonic also expressed confusion as to the meaning of the letter “I” (Ice and/or Immobilize).	RICE was modified to RI^2^CE, to account for the dual-meaning of “I”. The meaning of each letter was described in individual paragraphs of text.
**Iteration 3 English, n=4**	**Problems cited**	**Changes implemented**
	Users continued to find the background colouring of the “History of Hemophilia” animation inadequate.	Bright pictorial icons were added to improve the colour contrast of the animation (Figure 7).
**Iteration 4 French, n=6**	**Problems cited**	**Changes implemented**
	Three participants found the colouring of the videos too dark.	Budget constraints prevented aesthetic modifications to the videos.
	An English adage used on the site to help explain prophylactic factor use (Figure 8) did not bear the same meaning when translated into French.	Despite this nuance, French adolescents understood the content conveyed by the analogy, making modification unnecessary.

### User performance

User performance was evaluated through observation of navigation tasks. Navigation errors were defined as difficulties moving through or locating content or features [7]. Three first-round participants experienced navigation difficulties when asked to return to the home page. A “home” button was subsequently added to the navigation menu following the first round of testing and no further navigation errors were detected in subsequent iterations. Additionally, when the navigation menu was engaged, users’ location on the site was not obviously indicated on the menu. One participant in the first iteration experienced difficulty navigating between modules as a result. The menu was modified such that, when clicked on, the color of the heading text changed, making the user’s location on the site apparent. No further navigation errors of this nature were detected in succeeding iterations.

### User satisfaction

User satisfaction was assessed on the basis of themes emerging from the semi-structured interview: aesthetics of the user interface, content and features, functionality and sociability.

#### Aesthetics

Two subthemes arose under the subject of aesthetics: visual theme and layout. The majority of participants found the website banner and colors appealing, and overall, responded positively to the appearance of the site (Table 6). Videos on the site were technically treated with a filter to give a darker “graphic novel” look and were made more engaging through the insertion of B-roll footage (brief supplementary footage integrated between scenes). Three adolescents in the final round of testing suggested that the appearance of the videos were “too dark.” However, since only a few adolescents raised this concern and the logistics and cost of changing the appearance of all ten videos were not within budget, no changes to the look of the videos were made.

**Table 6:** Summary of user comments by theme

**Theme**	**Comments by adolescents**
**Aesthetics:** the theme, layout and visual appeal of the website	“I like how [the pictures] have the sort of comic bubbles to them…that part really stands out for me (age 16).”
	“Like the video was like of dark like a guy walking at night and really like really very dark like just the image is not what they’re saying…like it set not the right tone for the video cause it’s about adaptation (age 17).”
**Content and features:** the appeal of written text, animations, images and videos on the website	“It’s the first time I have seen videos on a hemophilia website (age 17).”
	“I thought it was pretty cool because they they had patients interviewed so you could kind of relate better to that (age 14).”
	“The animations are great, like the one about pain. You can really see what is going on (age 16).”
	“[The timeline] looks very black right now…If you got a bigger timeline um that took up more space on the page um we could add maybe a bit more color cause I mean it’s just black and black’s a plain color too right (age 17).”
**Understandability:** users’ level of comfort with the language, concepts and readability of the website	“Until you told me I actually didn’t know cause I was looking at the timeline and the mouse see it move from left to right or whatever (age 17).”
	“…kids younger might understand a lot better you know seeing the roads, how it relates to the blood vessels (age 17).”
	“It is a little too much text, but it is ok…I would read it because I want to know (age 16).”
**Comprehensiveness:** the breadth and completeness of the information offered on the website	“I think it really has everything (age 16).”
	“[It] gets into like lots of detail so if there is like any questions on your mind then there’s probably a subtitle for it that you can go and research on it or read about it (age 17).”
**Quality and credibility:** participants’ perceptions of the accuracy and trustworthiness of the website content [12]	“Most of the stuff that’s on the other websites…I call it like a bird’s eye view, like some else’s point of view looking in, but this [website] is like the teens, the parents, the doctors, everyone getting their [in]corporations…they’re like all voicing their opinion and stuff like that and you can see different point of views so it’s helpful (age 17).”
	“Somebody I know did this and I trust it more than just some other site that I just click on off Google or something. I know that actually actual people made this (age 16).”
	“[I like] how these [names] aren’t in bold cause on most sites the names of the people on it are like bold. These are just sort of there so that if they are like looking for it, you can find it but not really like standing out (age 16).”
**Sociability:** the ability of the program to facilitate user interaction with peers and health care providers [12]	“That [forum] would improve it…like a lot (age 13).”
	“[You can] connect with other hemophiliacs (age 13)”
	“If you want to ask a question but you can’t ask everybody…[then] you don’t have to tell who you are and where you live (age 16).”
**Intent for future use:**	“It gives me a lot more knowledge than I knew before… I probably would go home and go on the site, even after the study is done…because there’s some headings and some subtitles like that I haven’t seen before or like I’d like to know more in depth about (age 17).”
	“[Teachers] can come here and easily read it or look at it in detail (age 17).”
**Overall impression:**	“I thought it was very very well done – it was appealing the pictures and the videos and… always kept me intrigued…I like how it just put the information out there and you could just, you know, read and learn more about it (age 13).”
	“Most sites aren’t really like this…it would be a lot easier to understand if I had a website like this to look at (age 16).”
	“When I was young I would come to the hospital all the time and whatever and the nurses would be talking and then sometimes you know when people are speaking and you just nod your head and you’re just like yeah yeah yeah yes and then you don’t really understand?… it’s helpful because I guess next time or the times when you’re reading it you hear the word and it clicks in your head [you think] oh yeah back to [the website] (age 17).”

Participants were also asked their opinion on the website layout. Nearly all users stated that when accessing information online, they were discouraged when confronted with large blocks of text and preferred information to be presented in “chunks” or point-form. When asked about the amount and layout of text on the website, the majority of users felt that the amount of text was manageable and appreciated the accompanying images and animations that functioned to make the site “interesting and alive (participant, cycle 2, age 17).”

#### Content and features

Four subthemes were identified under the theme of content and features: understandability, comprehensiveness, quality and credibility (Table 6).

#### Understandability

**Figure 2:**
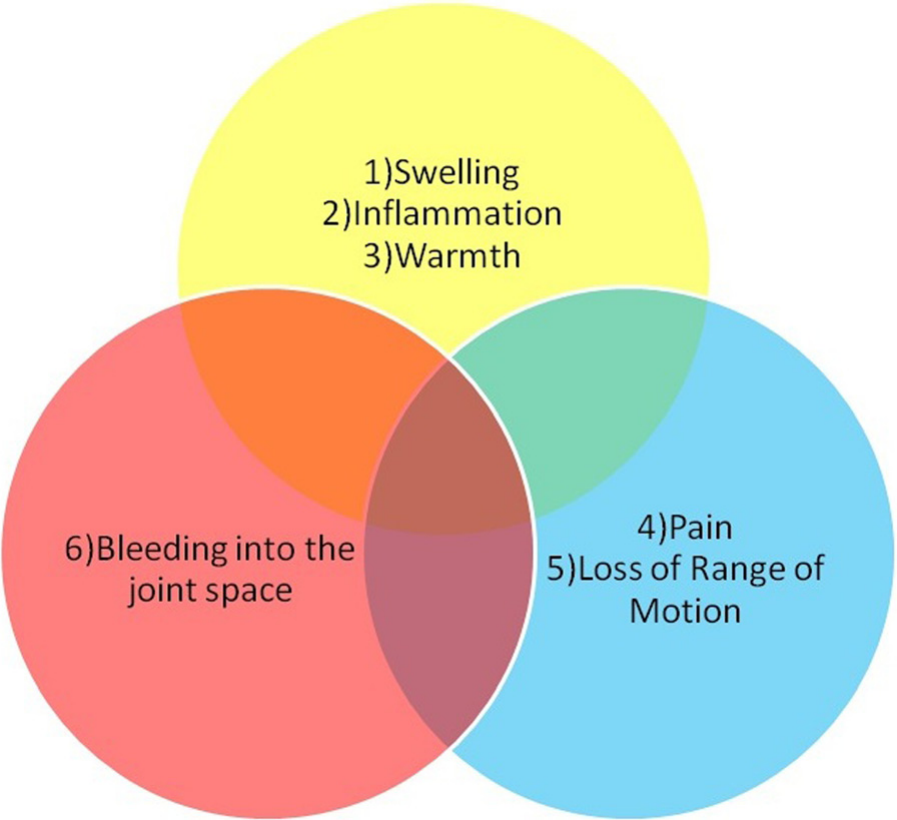
**Venn diagram before usability testing.** Graphic on the website illustrating the symptoms of arthritis and internal joint bleeds before usability testing was undertaken.

**Figure 3:**
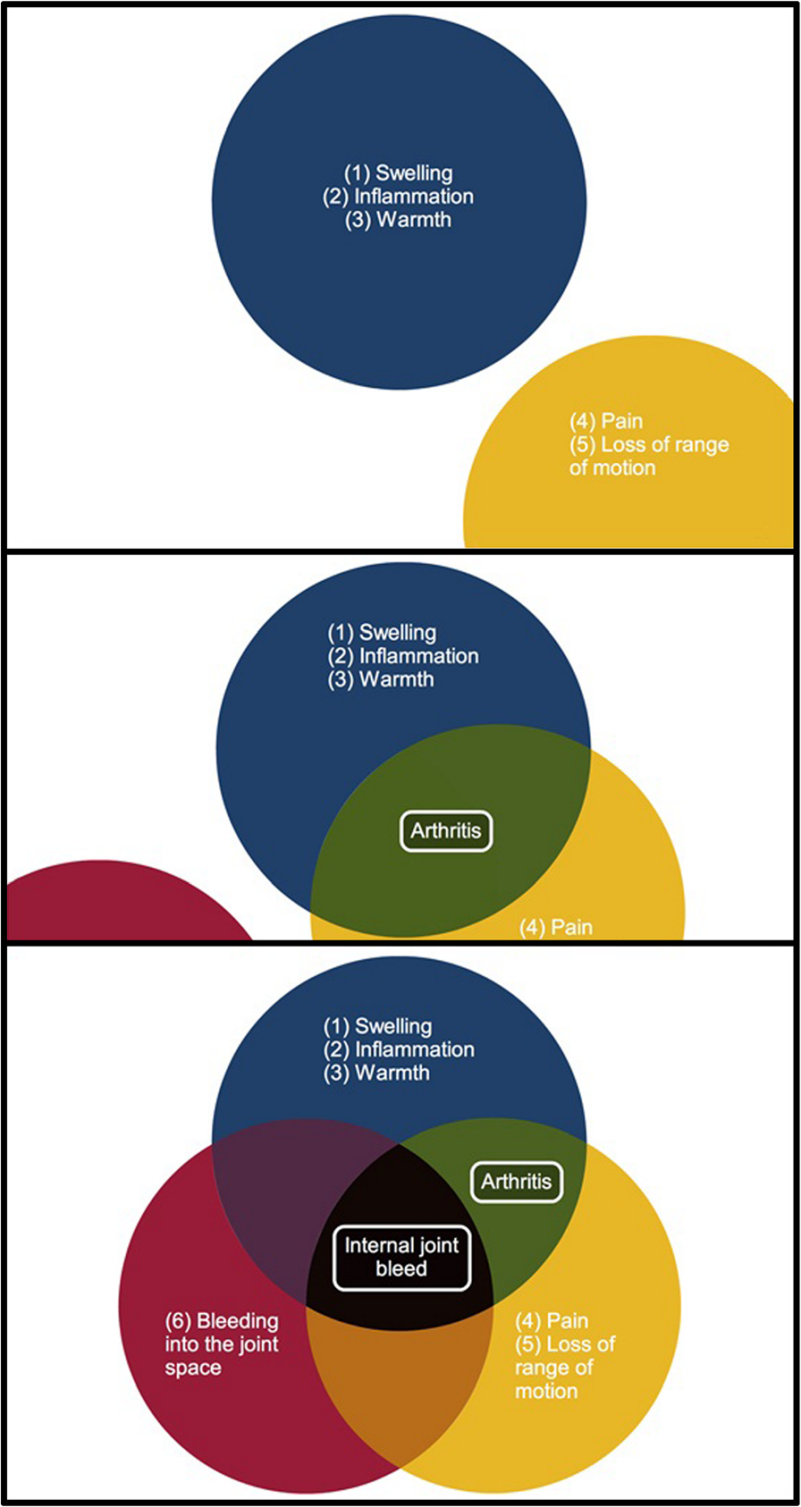
**Venn diagram after usability testing.** Following the first iteration of testing, three consecutive images were created to replace the previously single Venn diagram. Users found that these images more clearly conveyed the similarities and differences between arthritis and internal joint bleeds.

Users identified two images and one animation on the site that were difficult to understand. A Venn diagram was presented to draw a comparison between the symptoms of arthritis and joint bleeds,with the intent of emphasizing that blood in the joint space distinguishes a joint bleed from an arthritic joint (Figure 2). Two users in the first iteration did not understand that the intent of the diagram was to show the relationship between arthritis and joint bleeds by depicting their similarities and differences. The Venn diagram was subsequently broken down into multiple figures to first illustrate the symptoms of joint bleeds and arthritic joints as distinct sets, and then depict their joining (Figure 3). This example illustrates the danger of assuming familiarity with a concept like the Venn diagram, and that where possible, a simplistic approach should be used to convey information over conceptual representation. In subsequent iterations, no further concerns with the Venn diagram were identified.

**Figure 4:**
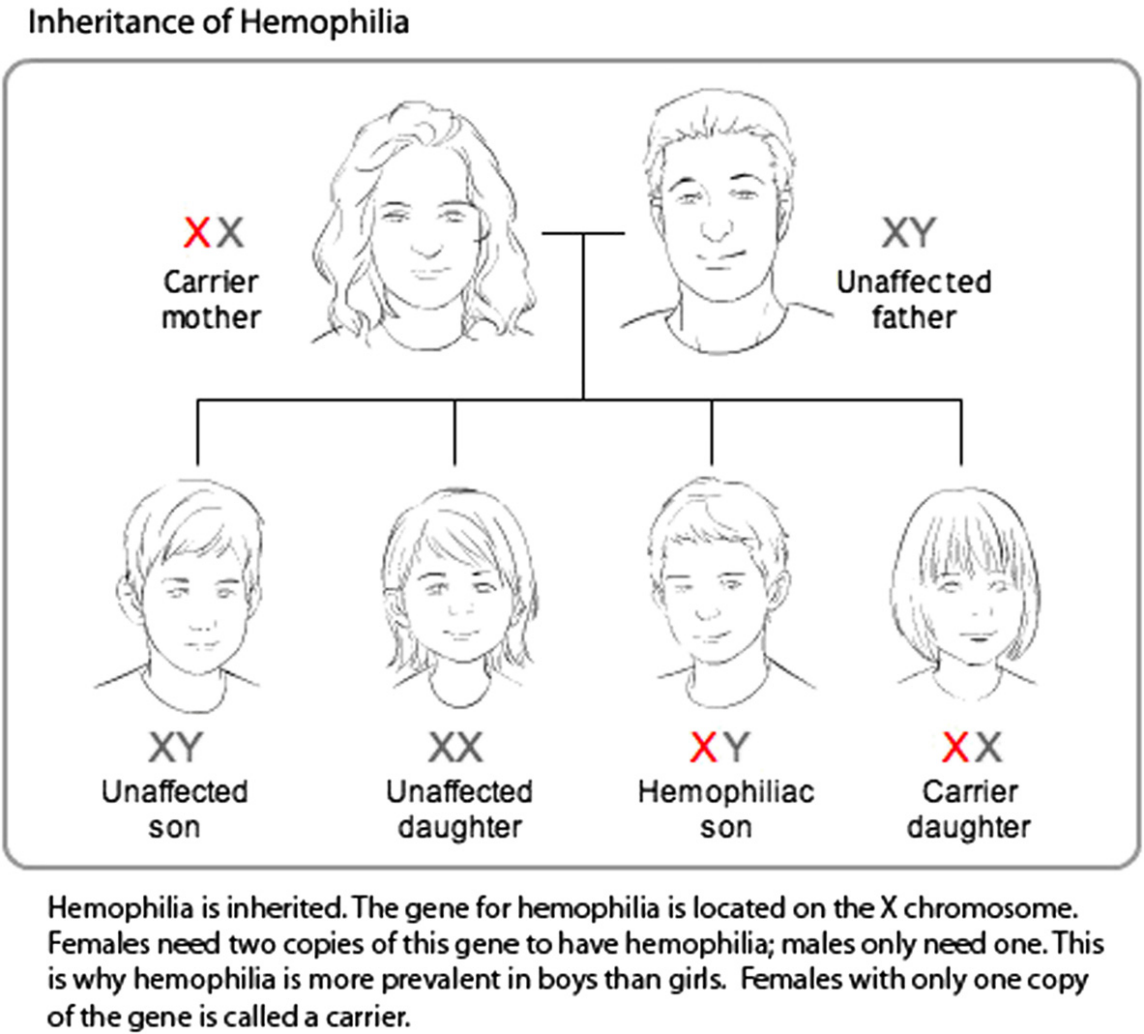
Image illustrating the heredity of hemophilia before usability testing.

**Figure 5:**
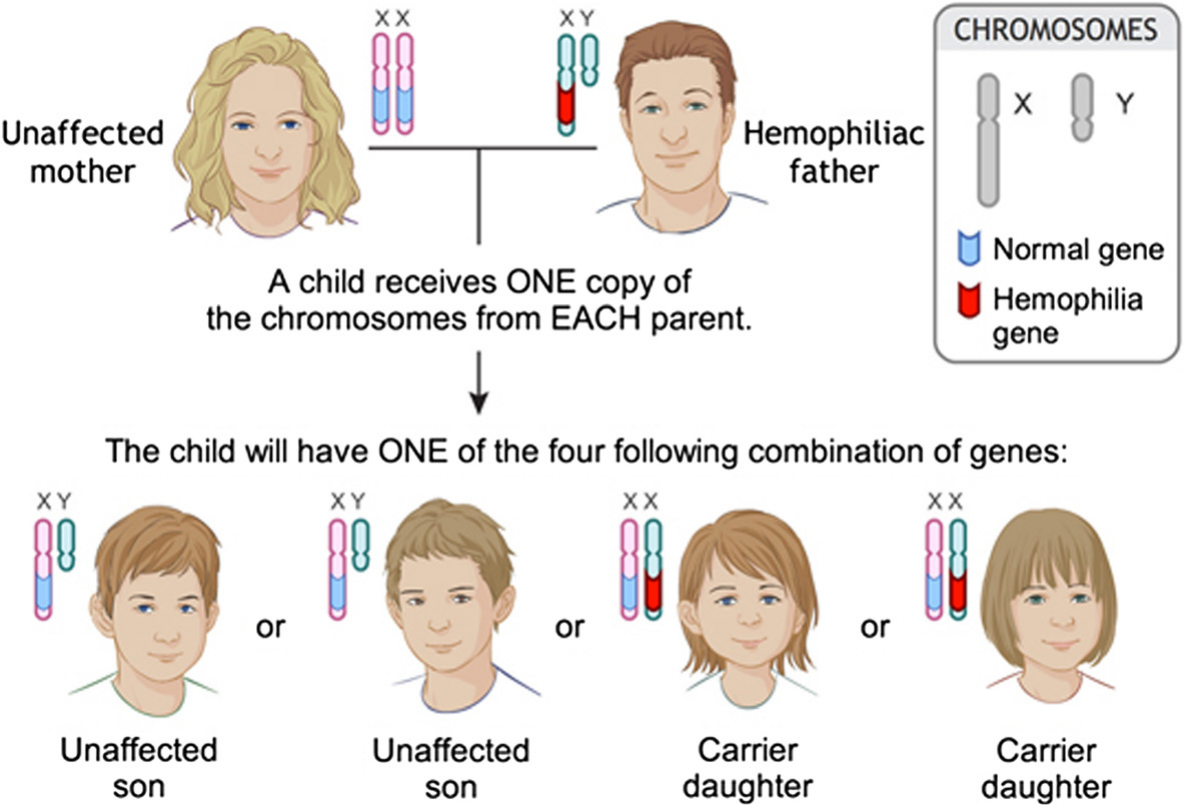
**Image illustrating the heredity of hemophilia after usability testing.** Following the first cycle of testing, maternal and paternal chromosomes were differentiated by color to demonstrate that offspring derive one sex chromosome from each parent.

The X-linked inheritance pattern of hemophilia is discussed on the site and accompanied by two images that illustrate all possible parental genotypes and the resulting probabilities of a child having or being a carrier for the disease (Figure 4). One participant in the first iteration had difficulty discerning why an affected father could not pass the trait on to his sons. Probing by the RA found this to be rooted in a lack of understanding of sex determination. This was rectified by differentially color-coding maternal and paternal chromosomes to demonstrate that offspring derive one sex chromosome from each parent (Figure 5).

**Figure 6:**
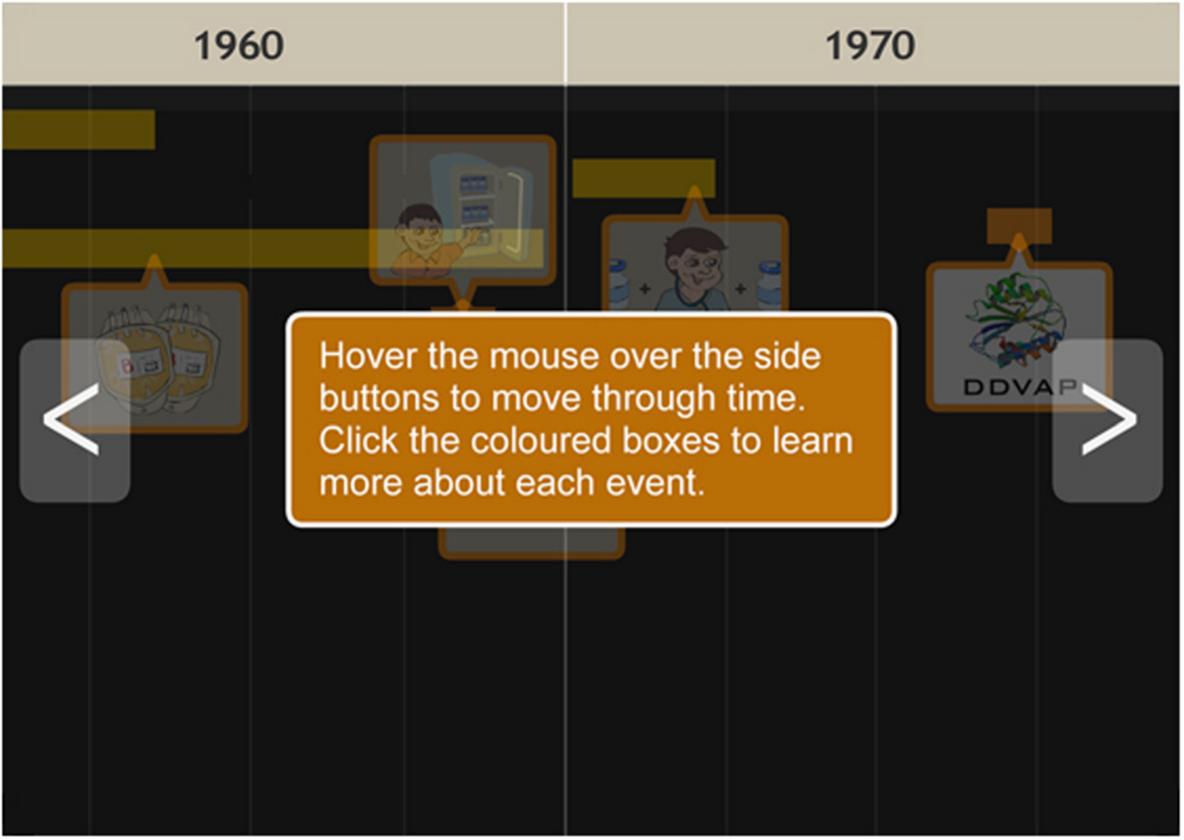
**History of hemophilia timeline animation after iteration 1.** Instructions were provided with the timeline to improve ease of use.

**Figure 7:**
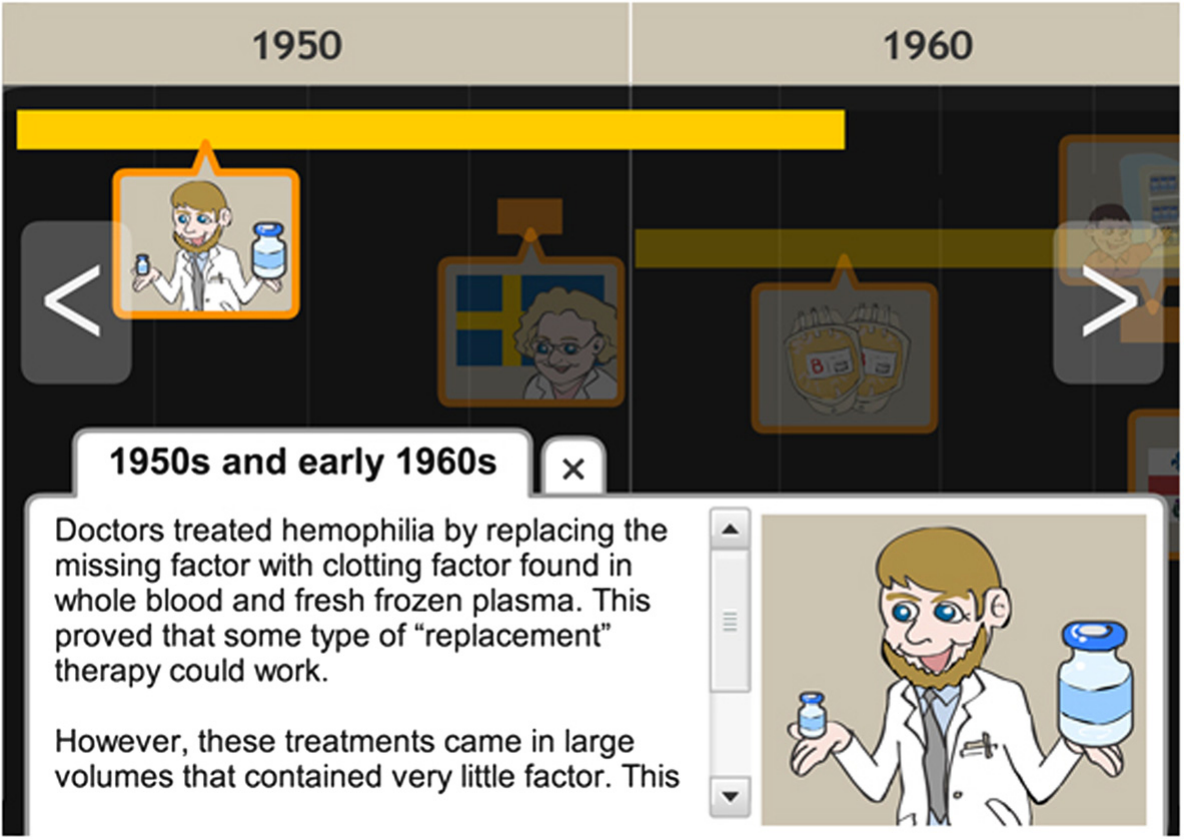
**History of hemophilia timeline animation after iteration 3.** Pictorial icons were inserted to improve ease of use and appeal.

The module on hemophilia management includes a “History of Hemophilia” animation that traces the progression of treatment options through time. In the first iteration, two adolescents were unable to intuitively use the timeline and found the coloring of the animation made it difficult to view in-laid bar icons. The animation was subsequently revised to include written instructions for use and the background color lightened (Figure 6). In the second iteration, adolescents still identified that the coloring did not provide satisfactory viewing. Two adolescents recommended the use of pictorial icons to remedy this. In response, water-marked images replaced the previously generic icons (Figure 7). No further concerns with the animation were identified in subsequent iterations.

One change was made to the presentation of text as a result of usability testing. To assist AWH in understanding hemostasis, an analogy to road repair was provided in paragraph form. One user stated that the analogy was difficult to follow in this form. Additionally, many participants stated they were not avid readers and felt intimidated by large amounts of text. Taking this into account, the text was reproduced as a table following the first round of testing (Table 7). Users in subsequent iterations found the table to be clear and easily understood. Overall, changes to content and features were found to enhance the understandability of the website.

**Table 7:** Road repair analogy used to explain hemostasis

**Your body**	**Road repair**
Blood vessels	Roads
Blood vessels wall	Road surface
Blood flow	Traffic flow
Platelets	Construction workers
Clotting factors	Road patch
Chemical signals	Radio communication used by construction workers. They use radios signals to request for materials needed to fix the damaged road.
Fibrin	Fibrin

#### Comprehensiveness

Adolescents in all iterations were pleased with the breadth of content presented. When asked if any content was missing, participants were unable to identify a topic of interest to them that had not been addressed.

#### Quality and credibility

The terms “quality” and “credibility” refer to participants’ perceptions of the accuracy and trustworthiness of the website content. Users in all iterations stated that they took quality and credibility into consideration when seeking health information online. When asked to comment on the quality of the website after viewing, users confidently stated it would rank among the top when compared to other hemophilia-specific information they had previously accessed online (Table 6).

The perceived credibility of the site proved to be an integral factor in quality rating. Adolescents stated that seeing health care professionals and peers in the video footage gave the website a personal feel and was indicative of the effort that had gone into tailoring the content to this specific patient group. One adolescent also stated that seeing the names and credentials of the authors at the bottom of each web page enhanced the credibility of the website, however the participant expressed uncertainty about the meaning of some of the abbreviations used. After hearing this sentiment echoed by other adolescents, abbreviated credentials were replaced with a brief lay sentence explaining each author’s professional role. Adolescents subsequently found this to be more understandable and further validated the contribution it made to the credibility of the site.

#### Functionality

Functionality refers to users’ satisfaction with the controls and operation of interactive features [7]. Seven adolescents were observed to seek a control to enlarge the size of the video. The addition of this feature was not supported by the content management system. No other concerns with functionality of the interface were identified by participants.

#### Sociability

Sociability refers to the Internet program’s capacity to facilitate user interaction with peers and health care providers and foster a sense of inclusion in a support network [7]. Adolescents overwhelmingly expressed desire for such features. Participants appreciated the opportunities that could be offered by a peer discussion board – a confidential domain in which to connect with other hemophiliacs, or conversely, the freedom to remain anonymous. Interestingly, adolescents expressed a unanimous preference for this feature to be termed a “forum” rather than “discussion board.” This underscored the influence of vocabulary on users’ perception of the website, and from a developers’ perspective, promoted greater attention toward the use of contemporary language. Adolescents also expressed desire for a place in which they could pose health-related questions to a trusted health care provider.

Following usability testing, these features were added. The forum will serve as an online space in which authorized users can anonymously post to other adolescent users. “Ask an Expert” provides the opportunity for users to privately post questions to an expert health care professional.

### Translation issues

**Figure 8:**
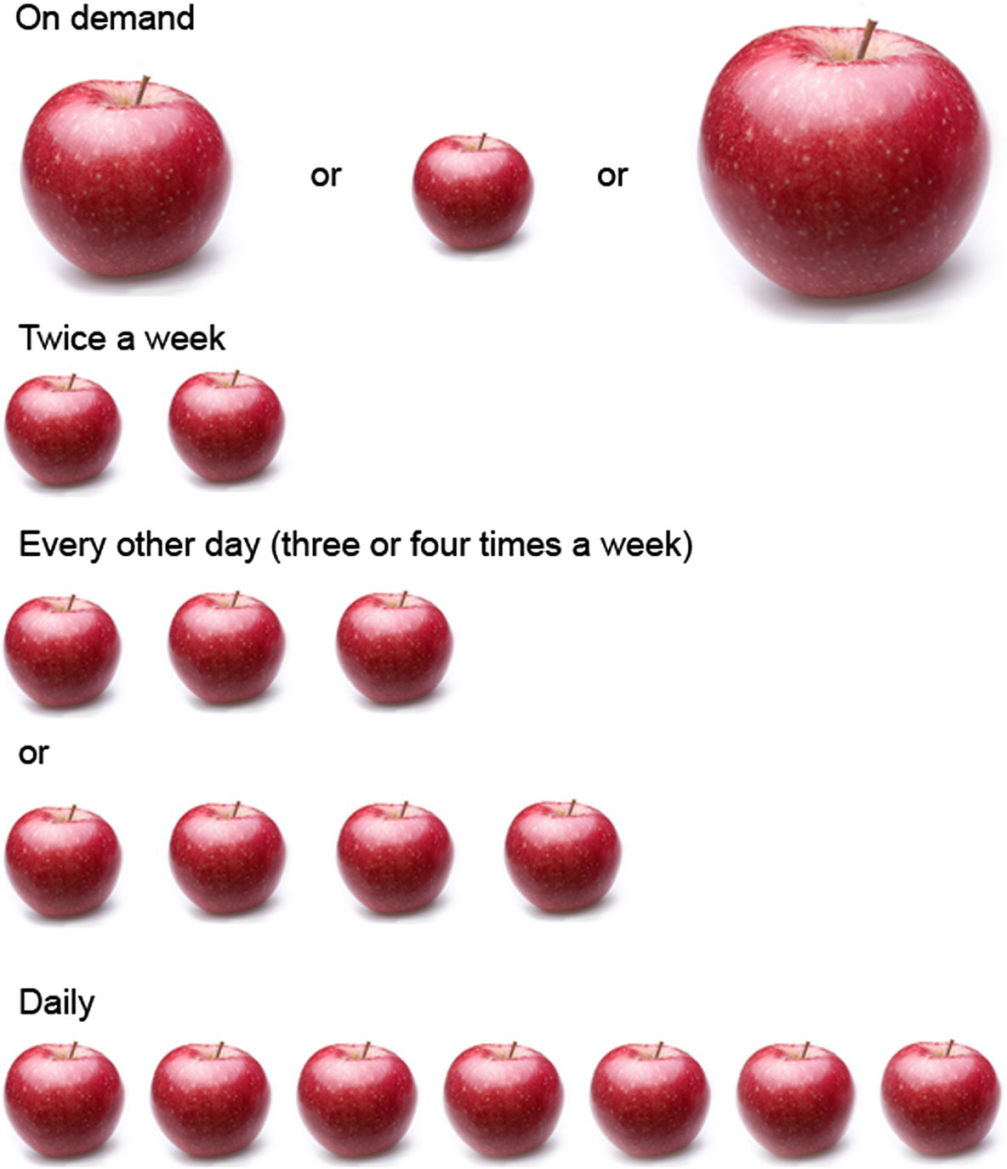
**“An apple a day keeps the bleed away”.** Analogy on the website using the English adage “An apple a day keeps the doctor away” to explain prophylactic therapy. One adolescent identified that this was not a familiar adage in the French language during usability testing.

Usability testing in French adolescents was undertaken to ensure the accuracy of translation. The module on hemophilia management adapted the adage “An apple a day keeps the doctor away” to read “A needle a day keeps the bleed away” and in French “Une pomme par jour éloigne le médecin pour toujours.” Equating an apple to a dose of factor, on-demand and prophylactic treatment regimens were explained and contrasted in the context of this analogy (Figure 8). While well-received among English-speaking users, one French adolescent identified that the adage on which the analogy was built did not bear the same traditional foothold in the French language. Despite this nuance, further probing by the RA found that the adolescent still understood the content conveyed by the analogy. No other French-speaking participants identified that the adage was unfamiliar to them. No additional culture-specific concerns were detected. As no significant changes were needed following this fourth cycle of testing, additional testing in French was not pursued.

### Overall impression

“Teens Taking Charge: Managing Hemophilia Online” was well-received by participants. Users were impressed with the visual assets, interactive features and breadth of content covered, and emphasized the program’s ability to remedy existing information gaps. The majority of participants stated that they would willingly use the program at home or would recommend it to friends, teachers or a significant other who wanted to learn more about hemophilia.

## Discussion

This study assessed user performance and satisfaction with a new Internet-based education program developed to assist AWH in self-management of their disease and transition to adult care. Web-based interventions are often designed by health professionals with minimal input from end-users during the development process. This carries with it the risk that the intervention will have little relevance and/or poor acceptability within the target population [20]. Findings from this study have reinforced that accurate, concise content written in plain language, use of visual media, attention to design elements and inclusion of social features are key elements of web-based interventions.

In addition, this study presents a specific example that suggests the importance of usability testing when translating websites for use in multiple languages. While testing in French was undertaken to ensure the accuracy of translation, it identified an analogy that was developed in English that failed to translate with the same meaning in French. Detection would not have occurred in the absence of testing. Concurrent development of content in the intended languages by a multilingual team may help prevent oversights. Whenever this is not feasible, it is important to test all programs following translation and prior to implementation in a new language. This finding also highlights the need to carefully consider cultural differences in the design process of multilingual programs and illustrates the role of usability testing in ensuring that content and visuals are culturally sound. Moore et al. showed the importance of usability testing when website developers hail from differing cultures, languages and backgrounds [21].

Additionally, this study identified that the quality and credibility of web-based content is an important consideration for many adolescents when presented with new information. Adolescents appreciated that authors and their credentials were identified on each web page and that these credentials were explained in lay terms. This contributed to the acceptability of the program and emphasizes that attention should be given to bringing the credibility of web-based interventions to user awareness. Programs such as the University of Washington’s “Connecting Youth to Quality Health Information” can be used to educate adolescents on how to assess the sites they are accessing for health information [22].

### Limitations

The limitations of this usability study must be considered. Firstly, the user-centred approach to usability testing has limits. The think-aloud method provides subjective data from a small number of participants. This made lead to concerns about validity and reliability of the data [23]. To limit the subjectivity, the usability testing team followed a pre-designed script for data collection. Although only a small number of teens participated, a representative sample based on severity of disease and age were recruited. This approach provided a rich source of data and met the criteria set forth by Nielson for usability testing sample size [16].

Another limitation was in the approach to recruitment. Participants were purposively selected for the initial three cycles at the English site and the final cycle at the predominantly French site. A more ideal approach would have been to include participants from both sites in each cycle, but this was not feasible due to distance and cost.

Finally, not all changes to the website proposed by adolescents were adopted due to software, funding and time constraints. Despite this, usability testing succeeded in bringing these concerns to investigators’ awareness. The proposed changes to the website that were not initially implemented will be re-considered in the future when updates are possible.

### Practice implications

The goal of any online health education program is to deliver health information in a manner that is accessible and appealing to the target population. An Internet-based program that neglects to consider the characteristics and needs of end-users incurs the risk of lacking relevance, accessibility and acceptability within the target population and may subsequently fail to meet its objectives. Systematic and methodological usability testing is a critical means of ensuring online health education interventions are tailored to the specific needs of the target population. Feedback acquired from participants through testing should guide improvements to the website prototype to enhance its functionality, understandability and appeal.

## Conclusions

This study illustrates the integral role of usability testing in the development of Internet-based health interventions and provides preliminary support for “Teens Taking Charge: Managing Hemophilia Online.” Errors encountered by users’ during observed navigation tasks informed changes that improved the ease of use of the website interface. User satisfaction was assessed based on adolescents’ feedback about the aesthetics, content and features, functionality and sociability of the website. Feasible changes were made to the prototype to improve user understanding and appeal. Usability testing was a fundamental step taken to ensure the program was relevant, cross-culturally valid and comprehensive.

The science of usability testing is developing at a fast rate. The majority of literature in the healthcare field involves usability testing of online health information systems, such as computerized order entry systems. The area of online patient education is developing on a parallel course, with increasing attention being paid to the development and testing processes. Guidelines from the US Department of Health and Human Services are available at http://www.usability.gov and provide an accessible primer to research-based website design and usability testing. Clinicians and patient educators should collaborate with experts in usability testing at the initial website planning stages to ensure appropriate and valid usability methodology is employed.

## Abbreviations

AWH: Adolescents with hemophilia; RA: Research assistant.

## Competing interests

The authors declare that they have no competing interests.

## Authors’ contributions

VRB conceived of the study, developed the study protocol, secured funding for website development, qualitatively analyzed interview transcripts and helped to draft the manuscript. AVW assisted with participant recruitment, helped conduct usability testing with adolescents, qualitatively analyzed interview transcripts and helped to draft the manuscript. DMI recruited study participants, conducted usability testing with adolescents and qualitatively analyzed interview transcripts. MW helped conduct usability testing with French-speaking adolescents and transcribed French interviews into English. VSB participated in the design of the study and assisted with participant recruitment. JNS participated in the design of the study, secured funding and acted as an advisor throughout the study process. All authors read, reviewed and approved the final manuscript.

## Pre-publication history

The pre-publication history for this paper can be accessed here:

http://www.biomedcentral.com/1472-6947/13/113/prepub

## Supplementary Material

Additional file 1 Part 1: Computer and Internet-Use Characteristics. Click here for file
