# Supplementary material for: The value of usability testing for Internet-based adolescent self-management interventions: “Managing Hemophilia Online”
Source: BMC Med Inform Decis Mak. 2013 Oct 4;13:113. doi: 10.1186/1472-6947-13-113 (PMC3856537; doi:10.1186/1472-6947-13-113)
Supplement: Additional file 1 — Part 1: Computer and Internet-Use Characteristics. [file 1472-6947-13-113-S1.doc]

**Additional file 1**

**Part 1: Computer and Internet-Use Characteristics**

Date of completion of form: ____________ (day/month/year)

1. How old are you? ________ years
2. What grade are you in at school:

7 8 9 10 11 12 CEGEP

1. What language do you speak most commonly at home?

English French other _______________

1. **a)** Do you use a computer at home? Yes No

**b)** If yes, do you have Internet access? Yes No

1. Do you use a computer at school? Yes No
2. How many hours do you use a computer each week? *(Circle ONE only)*

not at all 1-2 hours 4-5 hours

5-6 hours 6-7 hours >7 hours

1. Please circle the number that goes with how comfortable you feel using a computer.

1 2 3 4

not at all a little comfortable comfortable very comfortable

1. How many hours do you use **the Internet** each week (e.g. surf the net to find information)?

*(Circle ONE only)*

not at all 1-2 hours 4-5 hours

5-6 hours 6-7 hours >7 hours

1. Please circle the number that goes with how comfortable you feel using the Internet.

1 2 3 4

not at all a little comfortable comfortable very comfortable

**Part 2: Hemophilia Information**

1. What type of hemophilia do you have? A B not sure
2. What is the severity of your hemophilia?

mild moderate severe not sure

1. Are you currently on prophylaxis? Yes No not sure
2. **a)** Do you currently have an inhibitor? Yes No not sure

**b)** If not, did you have an inhibitor before? Yes No not sure

1. **a)** Have you had any unscheduled visits to the hemophilia clinic in the last 3 months?

Yes No

**b)** If yes, how many times and please explain reason for visit(s):

Number of visits:

Reason for visit(s):

1. **a)** Have you had any significant bleeds in the last 3 months?

Yes No

**b)** If yes, how many times has this happened and please describe the bleed(s):

Number of times:

Description(s):

1. **a)** Have you stopped taking any medications, changed dosage, or started any new medications in the past 3 months because of your hemophilia?

Yes No

**b)** If yes, how many times and please explain changes in your medication(s):

Number of times:

Explain change(s) in medication(s):

1. **a)** Have you started any new physical therapies (e.g. exercises, splints) in the past 3 months because of your hemophilia?

**b)** If yes, please specify which therapies:

1. **a)** Have you started any new psychological therapies (e.g. relaxation, cognitive-behavioural therapy) in the past 3 months because of your hemophilia?

**b)** If yes, please specify which therapies:

1. **a)** Have you started any new complementary or alternative therapies (e.g. herbal therapies, chiropractor) in the past 3 months because of your hemophilia?

**b)** If yes, please specify which therapies:

1. **a)** Have you missed any school because of your hemophilia in the past 3 months?

Yes No

**b)** If yes, please specify how many days:

1. **a)** Have you accessed any other websites about hemophilia and how to manage it, in the past 3 months?

Yes No

**b)** If yes, please specify how many times and which websites you visited:

Number of times:

Website(s) visited:

1. **a)** Have you contacted your healthcare team about your hemophilia and how to manage it during the past 3 months?

Yes No

**b)** If yes, please specify how many times and who you contacted:

Number of times:

Who was contacted:

1. **a)** Have you contacted any other teens with hemophilia on Facebook, MSN or any other social networking websites in the past 3 months?

Yes No

**b)** If yes, please specify how many times:
